# Th1 differentiation and function are inhibited in neonates following human metapneumovirus infection

**DOI:** 10.1093/jimmun/vkaf057

**Published:** 2025-04-25

**Authors:** Emma Brown, Jie Lan, Olivia B Parks, Cynthia S Hinck, Andrew P Hinck, John V Williams, Taylor Eddens

**Affiliations:** Department of Pediatrics, University of Pittsburgh School of Medicine, Pittsburgh, PA, United States; Department of Pediatrics, University of Pittsburgh School of Medicine, Pittsburgh, PA, United States; Medical Scientist Training Program, University of Pittsburgh School of Medicine, Pittsburgh, PA, United States; Department of Structural Biology, University of Pittsburgh School of Medicine, Pittsburgh, PA, United States; Department of Structural Biology, University of Pittsburgh School of Medicine, Pittsburgh, PA, United States; Department of Pediatrics, University of Pittsburgh School of Medicine, Pittsburgh, PA, United States; Institute for Infection, Inflammation, and Immunity in Children (i4Kids), Pittsburgh, PA, United States; Department of Pediatrics, University of Wisconsin School of Medicine and Public Health, Madison, WI, United States; Department of Pediatrics, University of Pittsburgh School of Medicine, Pittsburgh, PA, United States; Institute for Infection, Inflammation, and Immunity in Children (i4Kids), Pittsburgh, PA, United States

**Keywords:** animals—rodent, cells—Th1/Th2 cells, infections—viral, processes—tolerance/suppression/anergy, tissues—lung

## Abstract

Human metapneumovirus (HMPV) is a leading cause of lower respiratory tract infection in children accounting for 7% of acute care visits and hospitalizations. In particular, neonates and infants have worse outcomes with HMPV infection. The neonatal immune system is regulated to favor anti-inflammatory and tolerogenic responses compared to adults, including prior work demonstrating epigenetic factors in neonatal CD4^+^ T cells promoting Th2 formation rather than antiviral Th1 differentiation. To interrogate the neonatal immune response to HMPV, 4-to-6 day-old mice or adult 6-to-8 week-old mice were infected with HMPV. Neonates had a decreased Th1 population and increased Th2 and regulatory T-cell (Treg) populations compared to adults. Neonatal Th1 function, but not cell number, was restrained by surface PD-1 expression. To assess if neonatal Th1 formation was intrinsically inhibited after HMPV, neonatal and adult CD4s were transferred into immunocompetent or immunodeficient neonates. Both adult and neonatal CD4s demonstrated reduced Th1 differentiation in the immunocompetent neonates, but robust Th1 differentiation in immunodeficient neonates and immunocompetent adults, suggesting an extrinsic mechanism. Loss of neonatal Tregs led to increased Th1 differentiation after HMPV infection. Neonatal Tregs had increased TGF-β production compared to adult Tregs, and disruption of TGF-β signaling increased Th1 induction. These data demonstrate Tregs provide extrinsic regulation of Th1 formation in the context of respiratory viral infections, rather than an intrinsic limitation of neonatal CD4s. Collectively, these findings identify a nuanced neonatal response to respiratory viruses limiting Th1 formation and function.

## Graphical Abstract

**Figure:**
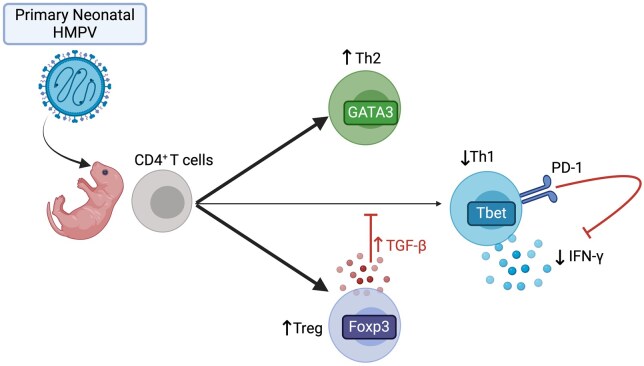

## Introduction

Lower respiratory tract infections (LRTIs) are the leading infectious cause of neonatal and infant mortality worldwide, resulting in an estimated 100 million cases and 740,000 deaths per year in children in the first 5 years of life.^1^ Respiratory viruses are a major contributor to the morbidity and mortality seen in young children. Three viral pathogens, respiratory syncytial virus (RSV), human metapneumovirus (HMPV), and influenza virus, result in approximately 57 million LRTIs annually in children less than 5 years old.^2–4^ RSV and HMPV infection is ubiquitous in this age range, as the majority of children demonstrate seropositivity by 2-to-3 years old.^5–7^ One prospective study demonstrated HMPV can be detected in approximately 7% of all children less than 5 presenting for respiratory illness or fever, with a higher rate of hospitalization in children in their first year of life.^8^^,^^9^ Infants born prematurely also have worse outcomes with HMPV infection, which further underscores the vulnerability of the youngest patients.^8^^,^^10^

One possible explanation for the severe nature of respiratory infection in young children is the differences in the developing immune system. While once thought to be in an immunosuppressed state due to immaturity, the immune system early in life is now recognized as regulated to favor anti-inflammatory and tolerogenic responses.^11–13^ We previously found using a neonatal model of HMPV that neonates were able to mount a robust HMPV-specific CD8^+^ T-cell response, but lacked the classical effector functions of antiviral CD8^+^ T cells seen in adult mice.^14^^,^^15^ PD-1, a marker of T-cell activation and inhibitory receptor, is expressed on neonatal CD8s and restrains production of these effector cytokines.^15^ Disruption of PD-1 signaling in neonatal mice resulted in a robust CD8^+^ T-cell response similar to adult mice, ultimately contributing to acute and long-term immunopathology after HMPV challenge.^15^ Collectively, these data demonstrate that neonatal CD8^+^ T cells are not incapable of mounting “adult-like” responses, but are actively regulated via PD-1 signaling.

Given the altered functions of CD8^+^ T cells in the neonatal HMPV, we sought to evaluate the CD4^+^ T-cell responses. Like CD8^+^ T cells, CD4^+^ T cells in neonates show disparate characteristics compared to adults. CD4^+^ T cells from neonates have a more restrictive T-cell receptor repertoire, are less likely to form memory cells, and have greater proliferative potential compared to adult responses.^16–21^ Moreover, generation of specific CD4^+^ T-cell subsets is altered in neonates, as neonatal CD4^+^ T cells more readily differentiate into Th2 cells and regulatory T cells (Tregs).^17^^,^^22^^,^^23^ Notably, Th2 cells are associated with allergy and asthma development due to the generation of a mucus and eosinophil-driven response, while Th1 cells are typically associated with antiviral immunity via IFN-γ production and facilitation of a cytotoxic CD8^+^ T-cell response. Epigenetic differences unique to neonates are the leading explanation for neonatal CD4^+^ T cell skewing, as Th2-promoting regions are more readily accessible in both murine and human cells.^24^^,^^25^ Neonatal Th2 cells also produce an abundance of IL-4, which can then directly signal to neonatal Th1 cells and induce apoptosis, further perpetuating a Th2-dominant environment.^26–28^

We sought to test whether the neonatal CD4^+^ T-cell response to respiratory viral infection was similarly constrained. Consistent with these prior studies, neonatal mice infected with HMPV mounted a skewed CD4^+^ T-cell response compared to adult mice, with a decreased antigen-specific Th1 response. PD-1 signaling restrained the production of IFN-γ by Th1 cells but did not limit Tbet^+^ CD4^+^ T-cell number. Strikingly, the lung milieu in immunocompetent neonates was capable of inhibiting Th1 formation in adoptively transferred neonatal and adult CD4^+^ T cells. Regulatory T cells in neonatal mice were responsible for constraint of Tbet^+^ CD4^+^ T cells and antigen-specific IFN-γ production via increased TGF-β production. These findings demonstrate the multifaceted regulatory processes that limit Th1 formation and function in the neonatal lungs after respiratory viral infection.

## Methods

### Mice and virus stocks

C57BL/6 mice (strain: 000664), *Rag1*^–/–^ mice (strain: 034159), B6.129S2-*Ighm^tm1Cgn^/*J (strain: 002288), B6.129P2-*Il4^tm1Cgn^*/J (strain: 002253), B6.129(Cg)-*Foxp3^tm^*^3^^*(Hbegf/GFP)Ayr*^/J (strain: 016958), and B6.SJL-*Ptprc^a^ Pepc^b^/*BoyJ (strain: 002014) were purchased from The Jackson Laboratory. *Pdcd1^–/–^* mice were obtained with permission from Tasuku Honjo (Kyoto University, Kyoto, Japan) and kindly shared by Karen Haas (Wake Forest University). Diphtheria toxin (0.3 µg/dose, MilliporeSigma) was administered every other day via i.p. injection. Breeding of 6-to-8 week-old mice of each strain was performed in the BSL-2 facility. Mice were monitored daily for signs of pregnancy. On day of life 4 to 6, neonatal mice were anesthetized with 3% isoflurane in a heated chamber and infected intranasally with 2.8 × 10^6^ PFU of HMPV strain TN/94-49 (genotype A2) in 10 µL sterile PBS, as described previously.^15^ Six-to-8-week adult mice were infected with 2.8 × 10^6^ PFU HMPV in 100 µL sterile PBS via orotracheal inoculation, as previously described.^14^^,^^15^^,^^29^ TN/94-49 was grown in LLC-MK2 cells and purified as previously described, while a lysate of uninfected LLC-MK2s was purified for use as a mock infection.^30^ Neonatal mice were euthanized via isoflurane anesthesia and decapitation, while adult mice were euthanized using CO_2_ asphyxiation. All protocols were approved by the University of Pittsburgh IACUC.

### Flow cytometry

Lung tissue was removed from the thoracic cavity, minced with scissors, and digested with DNase/collagenase for 1 h at 37 °C. The tissue was then passed through a 70-µm strainer and treated with RBC lysis buffer (ACK, Gibco, cat: A1049210) to generate a single cell suspension. Cells were then moved to a 96-well V-bottom plate and stained with Live/Dead Violet (1:1,000 in PBS, Invitrogen, cat: L34964A) for 15 min at room temperature. Cells were then washed ×2 in FACS, treated with Fc blockade (1:100 in FACS buffer, Tonbo, cat: 70-0161-M001) for 15 min at room temperature, and then stained for surface markers for 45 min at 4 °C (1.5 µL antibody/sample in BD Horizon Buffer, cat:566349; Table S1). Following 2 washes with FACS, cells were fixed with FOXP3 fix/permeabilization buffer overnight (Invitrogen, cat: 50-112-8857). After a wash in permeabilization buffer, cells were stained for intracellular markers for 1 h at 4 °C (2.5 µL antibody/sample in 1:1 mixture of BD buffer and fix/perm buffer). Cells were resuspended in FACS buffer and enumerated with counting beads, with gating strategies varying depending on the panel (Fig. S1).

For studies requiring peptide stimulation, a single cell suspension was plated in a 96-well U-bottom plate, treated with 10 µM of irrelevant peptide (NP311 from influenza or GP66-77 from Lymphocytic Choriomeningitis Virus [LCMV]) or an MHC class II–restricted immunodominant HMPV epitope (N217) in the presence of BFA/monensin and CD107-PE antibody for 5 h at 37 °C. Cells were then stained for Live/Dead and surface markers as above. Following 20 min in FOXP3 fix/perm buffer, cells were then stained for cytokine production (4.5 µL of antibody/sample).

For all conditions, samples were strained through nylon filters and run on a Cytek Aurora multispectral flow cytometer. Unstained cells from each experiment were fixed with 2% PFA and used for unmixing. FMO controls or isotype controls were used for gating placement and have been shown when applicable. Data analysis was performed with FlowJo (v10.8.1).

### ELISpots

Following generation of a single cell suspension from lung, 50,000 cells were plated per well in triplicate on an IFN-γ single color ELISpot plate (R&D systems, cat: EL485), with 10 µM of irrelevant peptide (GP66-77 from LCMV) or an MHC class II–restricted immunodominant HMPV epitope (N217).^31^ Treatment with anti-PD-L1 (10 µg/mL) was performed as described previously.^32^ The plate was incubated for 48 h at 37 °C and developed per manufacturer’s instructions.

### Cell purification and adoptive transfer models

Spleens were isolated from either adult or neonatal mice, followed by sequential filtering through a 70-µm strainer and 40-µm strainer with aMACS washes (498 mL of 1× PBS, 2.5 g BSA, and 2 mL of 0.5M EDTA). Following centrifugation, cells were resuspended in 80 µL aMACs and 20 µL CD4 negative selection beads (Miltenyi, cat: 130-140-454). Following a 5-min incubation on ice, anti-biotin beads (40 µL/sample) were added. Cells were then incubated for 10 min on ice and selected via primed magnetic LS columns (Miltenyi, cat:130-042-401), per manufacturer’s instructions. For transfer into *Rag1^–/–^* mice, CD90^+^ and CD19^+^ cell isolation was performed via positive selection per manufacturer’s instructions (Miltenyi). Cells were then washed with sterile PBS, enumerated, and resuspended in sterile PBS at a concentration of 2.0 × 10^6^ cells/30 µL. For adoptive transfer into neonates, the needle was inserted into the peritoneal cavity through the front side of the hind leg, as described previously,^33^ and 30-µL volume was delivered via a 30-g syringe. For transfer into adult mice, 2.0 × 10^6^ cells were resuspended in 200 µL PBS and administered intravenously through the tail vein via 27-g syringe.

### ImageStream

Cells were isolated and stained as per flow cytometry protocol with visualization via ImageStream as previously described.^34^

### TGF-β in vivo antibody/RER treatment

Mice were treated intraperitoneally as above with anti-TGF-β antibody (BioXcell #BE0057) or isotype control (BioXcell #BE0083) in 30 μL of sterile PBS via 30-gauge needle. RER was generated and purified, free of endotoxin, as described previously.^35^ Mice were injected with 100 µg/day in 30 µL PBS via i.p. injection. PBS was used as a vehicle control.

### Statistics

All data are displayed as mean ± SEM. All statistical analysis was performed using GraphPad Prism for Mac (v.10.1.1). Analyses with 2 groups were analyzed using an unpaired Student’s *t* test, while 3 or more groups were analyzed using a one-way ANOVA with Dunnett multiple comparisons. Experiments with 2 groups and multiple conditions were analyzed using a 2-way ANOVA with Tukey multiple comparisons. Significance was defined as *P* < 0.05 for all analyses.

## Results

### Neonatal mice have decreased Tbet induction in CD4^+^ T cells after HMPV infection

**Figure 1.**
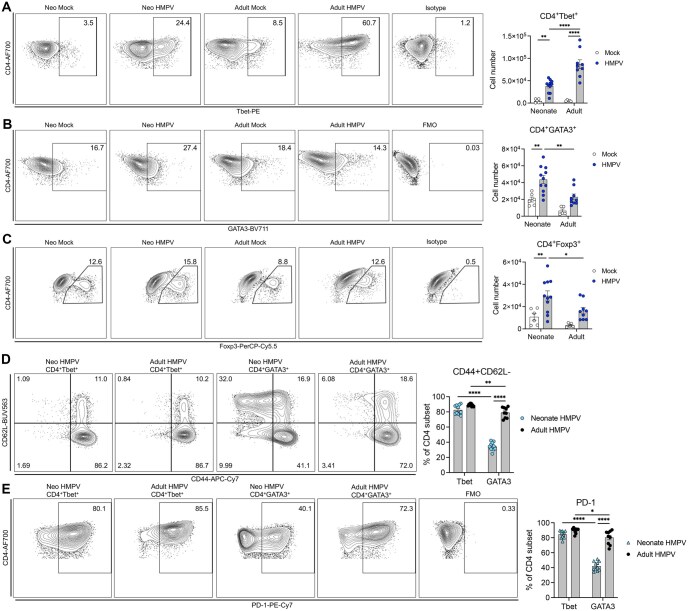
Neonatal mice have increased Th2 and Treg subsets compared to adult mice following HMPV infection. (A) Flow plots and enumeration of intracellular Tbet expression in lung CD4^+^ T cells following neonatal HMPV. (B) Flow plots and enumeration of intracellular GATA3 expression in lung CD4^+^ T cells following neonatal HMPV. (C) Flow plots and enumeration of Foxp3 expression in lung CD4^+^ T cells following neonatal HMPV. Isotype control or FMOs shown on far right panel. Neonate mock, *n* = 6; neonate HMPV, *n* = 11; adult mock, *n* = 5; adult HMPV, *n* = 9; representative of 2 to 3 experiments. Statistical analysis performed via 2-way ANOVA with Tukey multiple comparisons, *P < 0.05, **P < 0.01, ****P < 0.0005. (D and E) Proportion of activated (eg CD44^+^CD62L^–^) and PD-1-expressing CD4^+^ Tbet^+^ and GATA3^+^ cells following neonatal and adult HMPV. Neonate mock, *n* = 6; neonate HMPV, *n* = 11; adult mock, *n* = 5; adult HMPV, *n* = 9; representative of 2 to 3 experiments. Statistical analysis performed via 2-way ANOVA with Tukey multiple comparisons, *P < 0.05, **P < 0.01, ****P < 0.0005.

Neonatal (4 to 6 days old) and adult (6 to 8 weeks old) mice were infected with HMPV, and CD4^+^ T-cell transcription factor expression was assessed via intracellular staining at day 7 postinfection. Following infection, neonatal mice had a significantly increased number of CD4^+^ T cells expressing Tbet, the canonical marker of Th1s, compared to mock-infected neonates (Fig. 1A). Compared to adult mice, neonates had a significantly reduced number of Th1s (Fig. 1A). Neonates infected with HMPV, however, had increased Th2s (CD4^+^GATA3^+^) cells compared to both mock-infected neonates and HMPV-infected adult mice (Fig. 1B). Likewise, neonates infected with HMPV had increased Foxp3-positive Tregs compared to both mock-infected neonates and HMPV-infected adult mice (Fig. 1C). Neonatal mice favored Th2 cells at baseline, with a low Tbet/GATA3 ratio (Fig. S2A). After HMPV infection, neonatal mice had a Tbet/GATA3 ratio nearing 1, while adult mice had a 4:1 Tbet/GATA3 ratio (Fig. S2A). Phenotypic assessment of Tbet^+^ cells after neonatal and adult infection showed most cells were in the effector state (eg CD44^+^CD62L^–^), while neonatal GATA3^+^ cells were less likely to be activated (Fig. 1D). Collectively, neonates have relatively increased Th2 and Treg response with a decreased Th1 response compared to adult mice. Following adult and neonatal infection, Tbet^+^ T cells had robust expression of PD-1, an activation marker that functions as an inhibitory receptor on T cells (Fig. 1E). However, neonatal GATA3^+^ T cells had fewer cells expressing PD-1 compared to adult Th2s (Fig. 1E). Collectively, these data demonstrate neonatal Tbet^+^ T cells have similar activation profiles as adult cells, but are fewer in number in neonates.

### Neonatal Th1 function is restrained by PD-1

**Figure 2.**
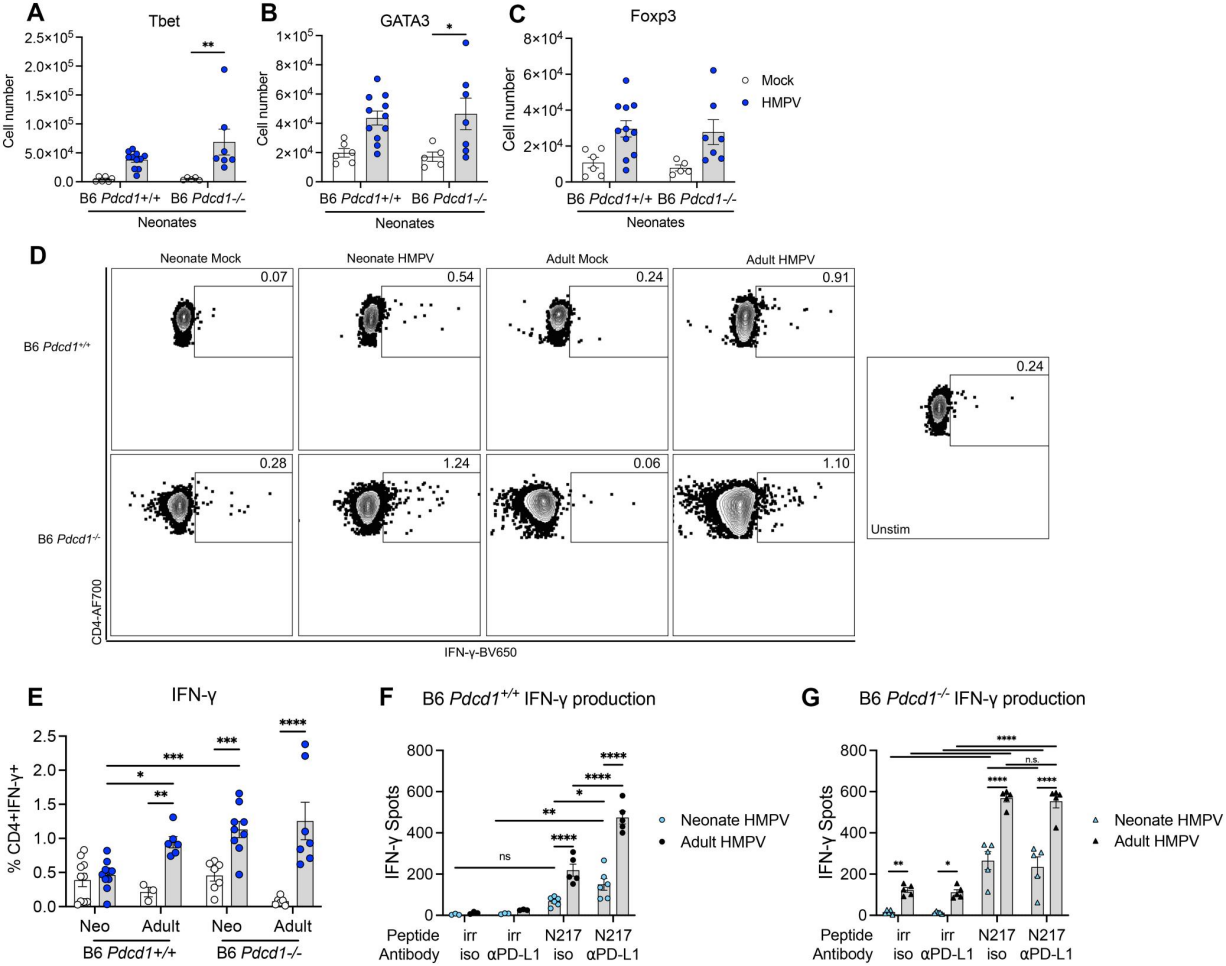
PD-1 inhibits Th1 following neonatal HMPV. (A–C) Enumeration of Tbet, GATA3, and Foxp3 expressing lung CD4^+^ T cells in mock-treated or HMPV-infected B6 *Pdcd1^+/+^* or B6 *Pdcd1^–/–^* neonates. *P < 0.05, **P < 0.01 by 2-way ANOVA. B6 *Pdcd1^+/+^* mock, *n* = 6; B6 *Pdcd1^+/+^* HMPV, *n* = 11; B6 *Pdcd1^–/–^* mock, *n* = 5; B6 *Pdcd1^–/–^* HMPV, *n* = 7; representative of 2 experiments. (D) Representative flow plots of intracellular staining for IFN-γ in mock treated or HMPV-infected B6 *Pdcd1^+/+^* or B6 *Pdcd1^–/–^* neonates or adults. Unstimulated cells shown on far right panel for comparison. (E) Quantification of IFN-γ intracellular staining. *P < 0.05, **P < 0.01, ***P < 0.001, ****P < 0.0005 by 2-way ANOVA with Tukey multiple comparisons, *n* = 3 to 10 for B6 *Pdcd1^+/+^* groups, *n* = 6 to 9 for B6 *Pdcd1^–/–^* groups. (F) ELISpot of IFN-γ-producing cells from the lung of B6 *Pdcd1^+/+^* at day 7 post–neonatal HMPV infection following ex vivo stimulation with an irrelevant peptide or MHC class II HMPV-immunodominant peptide (N217) ± anti-PD-L1 (10 µg/mL) or isotype antibody (10 µg/mL). Cells were stimulated for 48 h, and IFN-γ spot number was enumerated. (G) ELISpot of IFN-γ-producing cells from B6 *Pdcd1^–/–^* as described above. *P < 0.05, **P < 0.01, ****P < 0.0005 by 2-way ANOVA with Tukey multiple comparisons.

As PD-1 has been previously shown to inhibit the function of CD8^+^ T cells in neonatal and adult mice following HMPV infection,^14^^,^^15^^,^^36^ we next sought to elucidate the role of PD-1 on differentiation and function of the various CD4^+^ T-cell subsets. Neonatal mice lacking PD-1 (B6 *Pdcd1^–/–^* mice) had similar numbers of Tbet, GATA3, and Foxp3 expressing cells compared to wild type (B6 *Pdcd1^+/+^*) neonatal mice (Fig. 2A–C). Likewise, there was no statistically significant difference in Th1/Th2 ratio in *Pdcd1^–/–^* neonates compared to B6 neonates (Fig. S2B). However, *Pdcd1^–/–^* neonates had significantly increased CD4^+^ T-cell IFN-γ production compared to wild-type neonates (Fig. 2D and E). Detectable antigen-specific response was also observed in adult wild-type and *Pdcd1^–/–^* mice (Fig. 2D and E). To further demonstrate the role of PD-1 in limiting antigen-specific IFN-γ production, in vitro antibody blockade for a ligand of PD-1, PD-L1, was applied with conjunction with a HMPV-specific class II peptide stimulation (N217). In B6 with intact PD-1 signaling, N217 stimulation led to a modest increase in IFN-γ in neonates and a statistically significant IFN-γ in adults (Fig. 2F). Following blockade with anti-PD-L1, both neonates and adults had a statistically significant increase in IFN-γ spot number. In mice lacking PD-1 signaling, both neonates and adult mice had statistically significant increases in IFN-γ spot number after peptide stimulation (Fig. 2G). IFN-γ production was unaffected by anti-PD-L1 treatment in PD-1-deficient animals (Fig. 2G). In both *Pdcd1^+/+^* and *Pdcd1^–/–^* mice, adults had significantly more robust IFN-γ production compared to neonatal mice (Fig. 2F and G). These data demonstrate that wild-type neonates have fewer antigen-specific IFN-γ-producing cells compared to adults, while both neonate and adult IFN-γ responses are limited by PD-1 signaling.

### Neonatal CD4s are not intrinsically limited in Th1 differentiation

**Figure 3.**
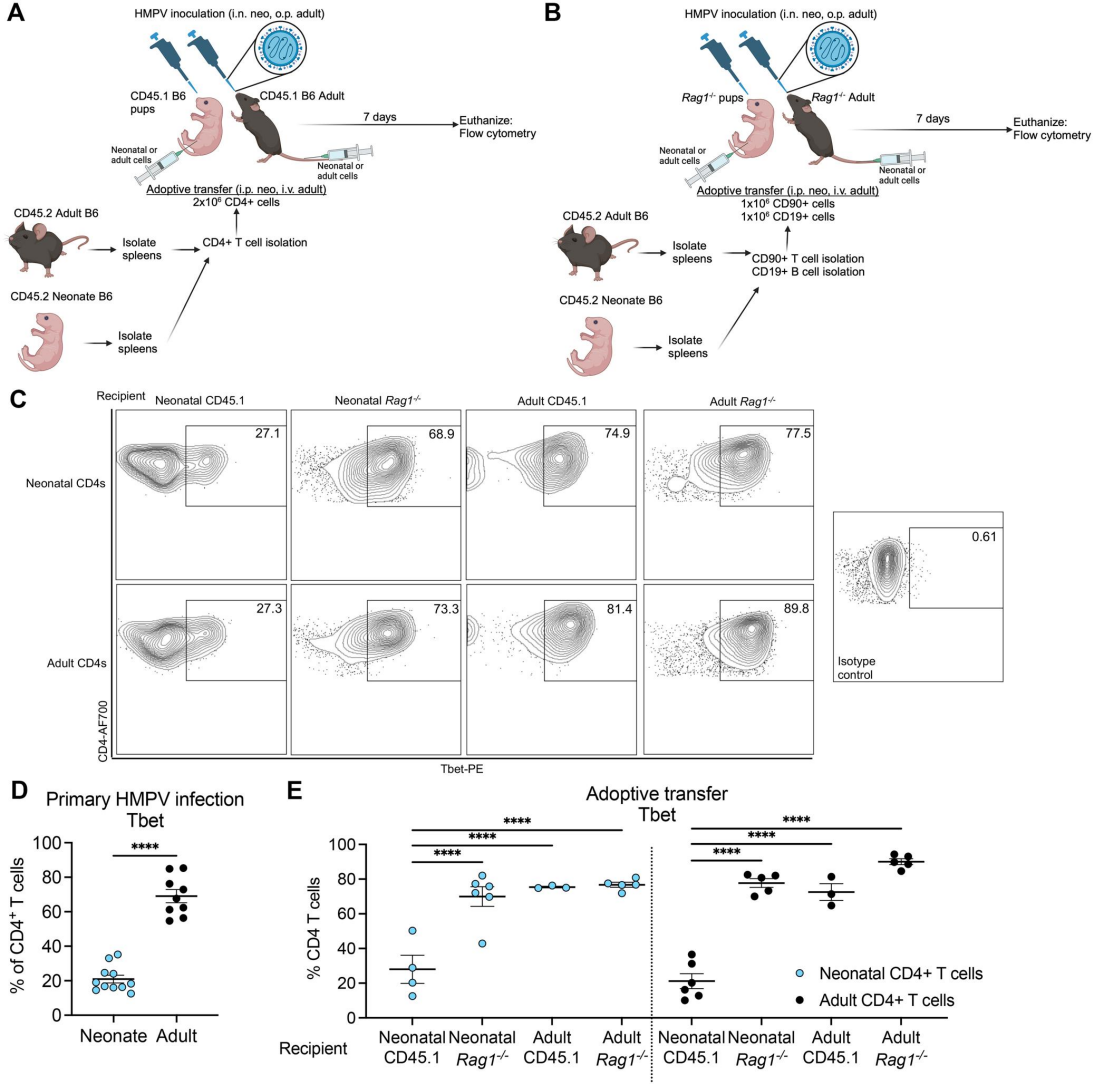
The immunocompetent neonatal lung environment suppresses Th1 differentiation in adult and neonatal CD4^+^ T cells. (A) Isolation of neonatal or adult CD4^+^ T cells with transfer into congenically labeled CD45.1 neonatal (i.p.) or adult (i.v.) mice. (B) Isolation of B and T cells from neonatal or adult donors with i.p. transfer into *Rag1^–/–^* neonates or i.v. transfer into adults. In both systems, mice are concomitantly infected with HMPV via intranasal inoculation and euthanized at day 7 postinfection for cellular analysis by flow cytometry. (C) Representative flow populations of Tbet expression in transferred lung CD4^+^ T cells following HMPV. Isotype control staining shown on far right. (D) Tbet induction in lung CD4^+^ T cells following primary HMPV in neonates or adults. ****P < 0.0005 by Student’s *t* test; neonate, *n* = 11; adult, *n* = 9; representative of 2 to 3 experiments. (E) Tbet induction in lung CD4^+^ T cells after adoptive transfer of cells into different recipients. ****P < 0.0005 by one-way ANOVA with multiple comparisons. *n* (from left to right): *n* = 4, 6, 3, 5, 6, 5, 3, 5. Representative of 1 to 2 experiments.

Prior studies suggest that epigenetic differences facilitate Th2 formation, which then prune Th1s.^23–27^ We next sought to determine if neonatal CD4s were intrinsically limited in Th1 differentiation following HMPV infection. To test this, we developed 2 murine models. To capture the context of the developing neonatal lung milieu, CD4^+^ T cells were isolated from B6 neonates or adults (CD45.2^+^) and adoptively transferred via i.p. injection into CD45.1 neonatal or adult recipients (Fig. 3A). In a second model, purified T and B cells from either a neonatal or adult donor were transferred into either a *Rag1^–/–^* neonatal or adult recipient (Fig. 3B). HMPV infection occurred concomitant with transfer in both models, and CD4^+^ T-cell differentiation was assessed at day 7 postinfection (Fig. 3A and B).

Neonatal CD4^+^ T cells transferred into a CD45.1 adult, *Rag1^–/–^* neonate, and *Rag1^–/–^* adult had robust Tbet expression as compared to cells transferred into neonatal CD45.1 recipients (Fig. 3C, top row). In comparison to primary HMPV infection (Fig. 3D), neonatal CD4^+^ T cells recapitulated “adult-like” levels of Tbet when in either a neonatal or adult *Rag1^–/–^* recipient environment (Fig. 3E). Adult CD4^+^ T-cell transfer into an adult CD45.1 or neonatal and adult *Rag^–/–^* recipients yielded similar Tbet expression (Fig 3C). Adult CD4^+^ T cells showed inhibited Tbet induction in the neonatal CD45.1 host, phenocopying neonatal cells in primary infection (Fig. 3B and C). In either *Rag1^–/–^* recipient, adult CD4^+^T cells differentiated into Th1s similar to levels seen in primary infection (Fig. 3B and C). In the absence of HMPV stimulation, this degree of Tbet upregulation was not observed in immunocompetent mice. However, Tbet was increased in neonatal cells in *Rag1^–/–^* adult mice consistent with a minor contribution of homeostatic proliferation in those recipients (Fig. S3). These data demonstrate 3 key points. First, neonatal CD4^+^ T cells are extrinsically and not intrinsically limited in forming Tbet^+^ cells. Second, the neonatal lung environment can limit Tbet upregulation by adult or neonatal CD4^+^ T cells. Third, the comparison between immunocompetent neonates and *Rag1^–/–^* neonates implicates lymphocytes in the process of limiting Th1 formation.

Given the elucidated role of lymphocyte populations, several candidate targets emerged: Th2 cells (which have been shown to contribute to Th1 apoptosis), B cells, and Tregs. To test these individual lymphocyte populations, we next assessed the role of the Th2 cytokine IL-4 using *Il4^–/–^* mice and B cells using *µMT*^–/–^ mice. The host’s cells served as the source of the neonatal CD4^+^ T cells in this system given. Chimerism of 1% to 2% adult donor cells was observed after adoptive transfer (Fig. S4A). In B6, *Il4^–/–^*, and *µMT*^–/–^ recipients, adult donor cells phenocopied the neonatal host cells, which displayed restricted Th1 differentiation (Fig. S4B). Th2 differentiation was reduced in both neonatal and adult cells in *Il4^–/–^* mice, while adult cells had a significant reduction in Th2 differentiation in *µMT*^–/–^ recipients (Fig. S4C). In all recipients, neonatal cells were more likely to form Tregs (Fig. S4D). These studies demonstrate that neither IL-4 signaling nor B cells are sufficient to restrain Tbet upregulation.

### Regulatory T cells are critical to restrain Th1 development in neonates

**Figure 4.**
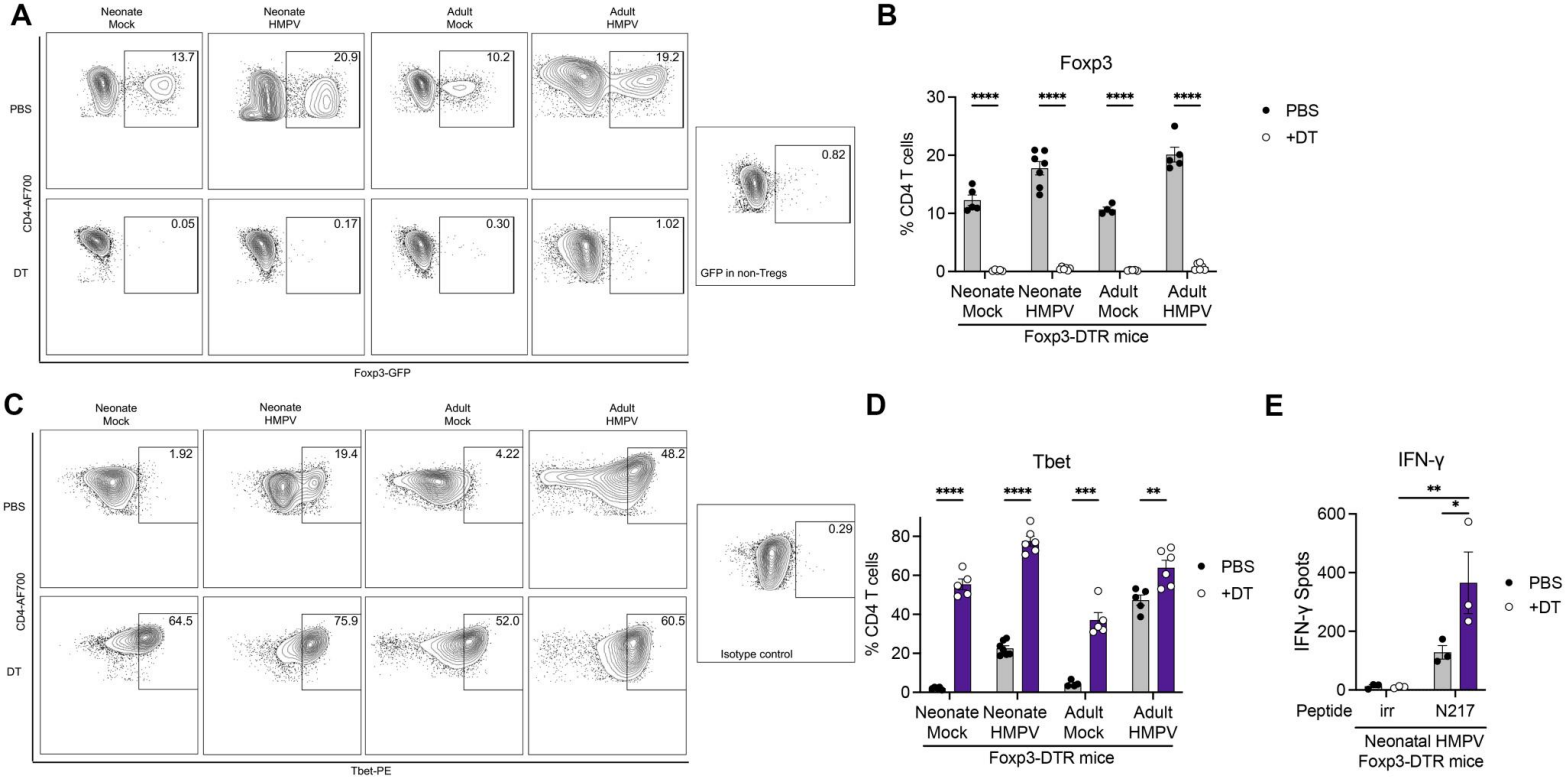
Loss of Tregs leads to increased Th1 induction post-HMPV. *Foxp3*-DTR neonates or adults were treated with 0.3 µg DT or equivalent volume of PBS every other day with HMPV or mock infection on day 0 and lung T-cell analysis via flow cytometry on day 7. (A) Representative flow panels of Foxp3+ T cells demonstrating successful depletion with DT treatment in all conditions. GFP-expression in Foxp3-APC negative CD4^+^ T cells shown for gating purposes. (B) Summary of Treg depletion with DT treatment in all conditions. (C and D) Tbet upregulation in mice was evident post–DT treatment in all conditions, including neonatal mice. **P < 0.01, ***P < 0.005, ****P < 0.0005 by multiple *t* tests, *n* = 4 to 7 with 2 experiments per condition. (E) IFN-γ ELISpot of cells isolated from the lung and ex vivo stimulation with irrelevant (irr) or HMPV (N217) peptide for 48 h. **P < 0.01, ****P < 0.0005 by 2-way ANOVA with Tukey multiple comparisons.

On the basis of these findings, we next assessed Tregs as a possible mechanism. *Foxp3*-DTR neonates or adults were treated every other day with diphtheria toxin (DT) following either HMPV or mock infection. Administration of DT successfully ablated Tregs in each condition (Fig. 4A and B). In HMPV-infected neonates, depletion of Tregs led to a marked increase in Th1 induction (Fig. 4C and D). A significant increase was also noted in HMPV-infected adult mice, although to a lesser magnitude than in neonates (Fig. 4C and D). Notably, both adult and neonate mock-infected animals also had significant increases in Th1 after depletion of Tregs (Fig. 4C and D). The number of antigen-specific cells was significantly increased in DT-treated, but not control, mice (Fig. 4E). Differentiation of neonatal T cells into Tbet^+^ IFN-γ-producing Th1s is therefore enhanced in the absence of Tregs.

### Neonatal tregs produce increased TGF-β

**Figure 5.**
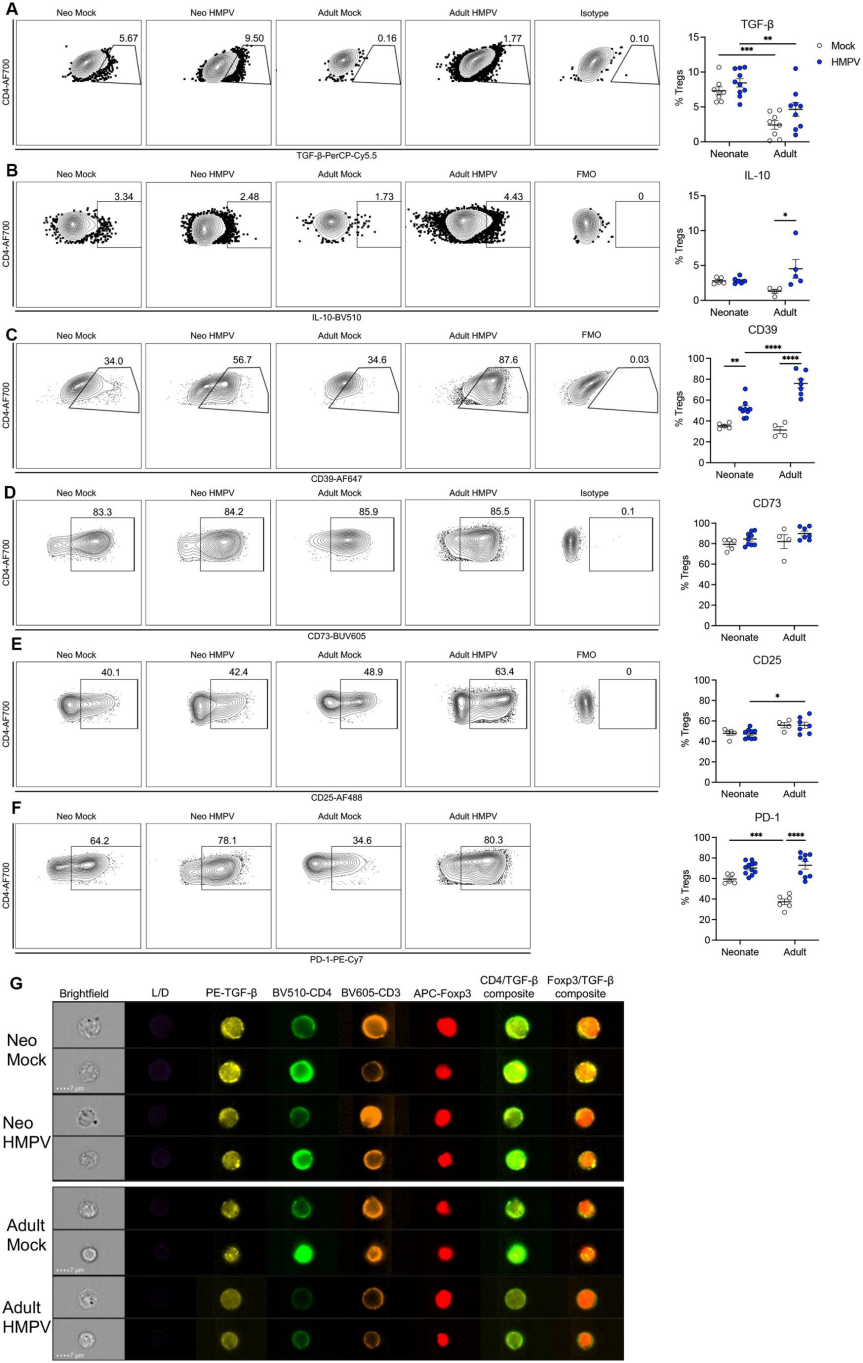
Neonatal Tregs produce increased TGF-β. (A) Intracellular cytokine staining for TGF-β in neonates or adult Tregs isolated from the lung following mock or HMPV infection, *n* = 8 to 10/group. (B) Intracellular cytokine staining for IL-10 in neonates or adult Tregs following mock or HMPV infection, *n* = 4 to 6/group. Large dots used on flow panels for display of cytokine production. (C and D) CD39 and CD73 surface expression on neonatal or adult Tregs, *n* = 4 to 9/group. (E) CD25 surface expression on neonatal or adult Tregs, *n* = 4 to 9/group. (F) PD-1 surface expression on neonatal or adult Tregs, *n* = 5 to 11/group. *P < 0.05, **P < 0.01, ***P < 0.005, ****P < 0.0005 by 2-way ANOVA with multiple comparisons, 2 to3 experimental replicates per group. (G) ImageStream analysis of TGF-β expression in neonatal or adult Tregs under mock or HMPV infected conditions. Neonatal groups had increased TGF-β signal.

The next question to address was which of the myriad Treg functions, such as consumption of IL-2 or ATP, inhibitory cytokine production, or expression of inhibitory receptors, limited Th1 differentiation.^37^ To that end, we assessed cytokine production (eg IL-10, TGF-β) and surface proteins (eg CD25, CD39, CD73, and PD-1) associated with Treg-mediated inhibitory functions.^37^ TGF-β was significantly elevated in both mock and HMPV-infected neonates compared to adults (Fig. 5A). IL-10 was unchanged in neonates with infection but was significantly upregulated in adults following HMPV infection (Fig. 5B). CD39, an ectonucleotidase associated with ATP cleavage, was upregulated with HMPV infection, but to a lesser extent in neonates (Fig. 5C). CD73, an additional ectonucleotidase, was similarly expressed in all groups (Fig. 5D). Adult Tregs showed a modest but significant increase in CD25 after HMPV infection compared to neonatal Tregs (Fig. 5E). PD-1 was more abundant on neonatal Tregs at baseline but was similar in neonates and adults after infection (Fig. 5F). As TGF-β was the most prominent difference in neonatal Tregs, we confirmed this difference using ImageStream analysis. Neonatal Tregs had increased TGF-β signal (yellow) in mock and HMPV-infected states compared to adults (Fig. 5G).

### Disruption of TGF-β signaling in neonates increases Th1 differentiation

**Figure 6.**
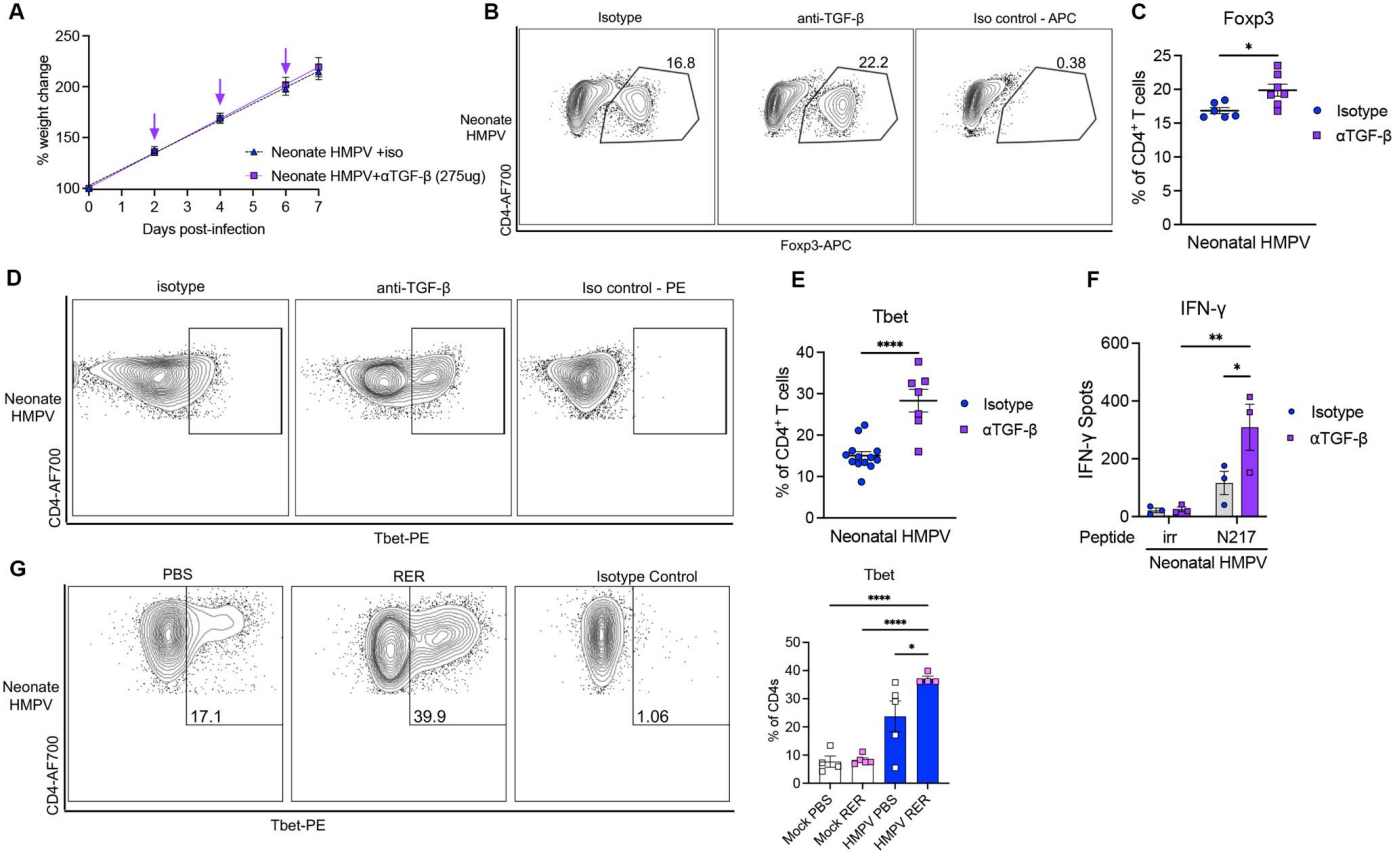
Disruption of TGF-β signaling increased Th1 abundance following HMPV infection. (A) Neonatal mice were treated with isotype or anti-TGF-β antibody every other day (as indicated by arrows) with euthanasia at day 7 post–HMPV infection. There was no significant difference in weight gain with isotype or anti-TGF-β antibody treatment. (B and C) Representative flow panels and summary of Foxp3^+^CD4^+^ T cells after isotype or anti-TGF-β antibody treatment *n* = 6 to 7/group, *P < 0.05 by *t* test. (D and E) Representative flow panels and summary of Tbet^+^CD4^+^ T cells after isotype or anti-TGF-β antibody treatment, *n* = 7 to 13/group, representative of 3 experiments, ****P < 0.0005 by *t* test. Isotype control for PE staining shown on far right. (F) IFN-γ ELISpot demonstrating increased spot number following N217 stimulation in mice treated with anti-TGF-β antibody (*n* = 3/group). *P < 0.05, **P < 0.01by 2-way ANOVA with multiple comparisons. (G) Neonatal mice were treated with PBS or RER (100 µg/day) following mock or HMPV infection. Representative flow plot and summary of Tbet induction, *P < 0.05, ****P < 0.0005 by 2-way ANOVA with multiple comparisons, *n* = 4 to 5/group, one experimental replicate.

To assess the role of TGF-β on T-cell differentiation, we treated neonatal mice with an anti-TGF-β monoclonal antibody during the duration of HMPV infection. Treatment with anti-TGF-β did not impact weight gain in neonates (Fig. 6A). Treg frequency significantly increased despite blockade of anti-TGF-β treatment (Fig. 6B and C). Tbet expression was significantly increased in lung CD4^+^ T cells after treatment with TGF-β blockade (Fig. 6D and E). Lung cells from anti-TGF-β–treated mice had increased IFN-γ spot frequency following stimulation with N217 (Fig. 6F). As a complementary approach, mice were treated with a soluble trivalent TGF-β trap known as RER, which has been shown previously to increase antagonistic action on TGF-β signaling compared to other methods.^35^^,^^38^^,^^39^ Treatment with RER in HMPV-infected neonates resulted in an increased Tbet frequency (Fig. 6G). RER treatment in uninfected neonates showed no effects on Tbet induction in the absence of infection (Fig. 6G). Collectively, these data demonstrate inhibition of TGF-β signaling leads to increased Tbet^+^ and IFN-γ-producing cells consistent with Th1s in the setting of a respiratory viral infection.

## Discussion

The immunologic tone established in the neonatal lung is strikingly different from that in adult mice.^11^^,^^40^ Neonates generally limit inflammation and promote tolerance when compared to adults. This is evident when examining the CD4^+^ T-cell compartment, as neonatal mice have been shown to favor Th2 and Treg development at the expense of canonical antiviral Th1 formation. Here, we show that neonatal CD4^+^ T cells are capable of generating Th1s in an adult microenvironment or immunodeficient neonate. Further, neonatal CD4^+^ T-cell production of IFN-γ is restrained by PD-1, while Th1 formation is actively inhibited by Tregs and TGF-β signaling.

The mechanisms of Th2 skewing in neonates and infants have long been of interest, as Th2-driven conditions like eczema, allergy, and asthma often emerge early in childhood.^41–43^ Th2 skewing has been attributed to intrinsic factors unique to neonatal CD4^+^ T cells, including epigenetic modifications to the genome and pruning of Th1s via IL-4-driven apoptosis.^23–27^ Consistent with this literature, we show that neonatal mice have increased GATA3^+^ and decreased Tbet^+^ CD4^+^ T cells with primary HMPV infection compared to adult mice. However, neonatal CD4^+^ T cells show robust differentiation into Th1s in response to HMPV when transferred into adult mice (regardless of immune status) and immunodeficient neonates. These findings strongly suggest that intrinsic factors, such as epigenetic changes, are not sufficient to limit the formation of Th1s. Moreover, the ability of the neonatal lung milieu to suppress the formation of Th1s by adult CD4^+^ T cells further establishes an extrinsic process as the mechanism.

There are a number of reasons why the neonatal lung milieu may be different and more immunosuppressive than the adult lung. First, the lung is still actively developing; postnatal alveolarization occurs for the first 4 weeks of life in mice and 1 to 2 years in humans, increasing the lung surface area 6-fold.^44^ This highly-orchestrated process requires activation of a number of distinct signaling pathways and may include temporally regulated influx of various immune cells (eg eosinophils).^45^^,^^46^ Second, neonates also have different diets (eg breastfed vs. chow) and are impacted by maternal influences (hormone, prior exposures). Another consideration is the acquisition and maturation of a stable lung microbiome, which can influence tolerance. The presence of a microbiome was required for maturation of Tregs and upregulation of PD-L1 in the lung, which then facilitated tolerization to an allergenic exposure.^47^

The role of Tregs in establishment of systemic tolerance is well described, in part due to the inborn error of immunity resulting from lack of functional FOXP3, known as IPEX (Immune dysregulation, Polyendocrinopathy, Enteropathy, and X-linked syndrome).^37^ In IPEX, Tregs are unable to suppress formation of effector CD4s, resulting in systemic activation and autoimmunity.^48^^,^^49^ IPEX patients have phenotypic features consistent with Th2-driven immunity (eg eczema, eosinophilia, increased IgE). Similarly, several studies have demonstrated that loss of Treg function in patients and preclinical models leads to a dominant peripheral Th2 response.^50–52^ These findings support the notion of a baseline Th2 bias within neonatal CD4^+^ T cells, which becomes unleashed in the absence of Treg-mediated suppression. In the presence of a respiratory viral infection, however, Th1 differentiation is also actively inhibited by Tregs in neonatal mice. This discrepancy may indicate that the role of Tregs may differ depending on the specific combination of site and stimuli.

While the current study establishes the role of TGF-β and PD-1 in limiting antiviral Th1-cell number and function, respectively, both molecules are critical in the developing lung. TGF-β and its isoforms are temporally regulated during the antenatal period, with perturbations resulting in aberrant lung development.^53–58^ Alterations of TGF-β isoform expression have also been implicated in postnatal murine models of bronchopulmonary dysplasia, a chronic lung condition in premature infants characterized by abnormal lung architecture.^53^^,^^59–63^ In contrast to TGF-β, the inhibitory function of PD-1 is critical in limiting immunopathology. One child with complete genetic loss of PD-1 signaling ultimately succumbed to pulmonary autoimmunity.^64^ In the context of neonatal respiratory viral infection, global loss of PD-1 signaling resulted in increased acute weight loss and chronic immunopathology with abnormal lung architecture.^15^ The inability of the neonatal lung to tolerate disruptions in TGF-β or PD-1 signaling without long-term consequences emphasizes the delicate nature of the developing lung and the need for tight immune regulation to mitigate damage.

PD-1 signaling has been implicated in Th1 biology in a variety of disease states. In tumor-infiltrating CD4^+^ T cells in humans, PD-1 signaling suppressed Tbet and limited IFN-γ production through SHP-2.^65^ In murine experimental autoimmune encephalitis, disruption of PD-1 signaling led to enhanced IFN-γ production.^66^ In an infectious model, a subset of *Mycobacterium tuberculosis*-specific CD4^+^ T cells upregulated PD-1; PD-1^+^CD4^+^ T cells in this system had decreased cytokine production, but increased proliferative capacity.^67^ In skin, PD-1 blockade in antigen-specific CD4^+^ T cells led to sustained effector cytokine production.^68^ The current studies complement this body of literature by demonstrating the role of PD-1 in limiting neonatal CD4^+^ T-cell production of IFN-γ.

The current work has limitations and leads to additional questions that could inspire future studies. One limitation is determining whether TGF-β signals directly to CD4^+^ T cells within the lung. TGF-β could contribute to this process in a distant site (eg lymph node) or could indirectly affect CD4^+^ differentiation via communication through intermediary cell populations. Likewise, understanding the downstream effects of TGF-β on specific cell types could inform targets of either promoting or limiting Th1 formation in neonates. The role of PD-1 on specific CD4^+^ T-cell subsets, such as Tregs vs. Th1s, and the lack of PD-1 on Th2s could be investigated in follow-up studies using genetic approaches. The previously established role of Th2-driving epigenetic changes in neonatal CD4^+^ T cells, and how these may be overcome in the setting of an infection, could be explored. While the current study evaluated transfer of adult cells into *Il4^–/–^* mice, these studies were limited by lack of transfer of neonatal cells. The role of Th2 cells and the cross talk with Th1s could be further explored using various knockout mice in our models. Collectively, the current study demonstrates the novel concept that neonatal CD4^+^ T cells are not intrinsically limited in developing a robust Th1 response. Rather, Th1 formation and function in neonates is inhibited by Treg production of TGF-β and PD-1, respectively, demonstrating a nuanced immune response within the developing lung.

## Supplementary Material

vkaf057_Supplementary_Data

## Data Availability

The data underlying this article are available in the article and in its online supplementary material.
