# Supplementary material for: Th1 differentiation and function are inhibited in neonates following human metapneumovirus infection
Source: J Immunol. 2025 Apr 25;214(7):1827–38. doi: 10.1093/jimmun/vkaf057 (PMC12311372; doi:10.1093/jimmun/vkaf057)
Supplement: vkaf057_Supplementary_Data [file vkaf057_supplementary_data.pdf]

Th1 differentiation and function is inhibited in neonates following respiratory viral infection

Emma Brown[#], Jie Lan[#], Olivia B. Parks[†], Cynthia S. Hinck[‡], Andrew P. Hinck[‡], John V. Williams[#,§,&], Taylor Eddens[#,§,*]

[#]University of Pittsburgh School of Medicine, Department of Pediatrics, Pittsburgh, PA, USA
[†]University of Pittsburgh Medical Scientist Training Program, Pittsburgh, PA, USA
[‡]University of Pittsburgh School of Medicine, Department of Structural Biology, Pittsburgh, PA, USA
[§]Institute for Infection, Inflammation, and Immunity in Children (i4Kids), Pittsburgh, PA, USA
[&]University of Wisconsin School of Medicine and Public Health, Department of Pediatrics, Madison, WI.

Running title: Inhibition of neonatal Th1s after respiratory viral infection

Corresponding Author:

*Taylor Eddens

4401 Penn Avenue

Rangos Research Building 9128

Pittsburgh, PA 15224

Phone: 724-272-9208

Email: taylor.eddens@chp.edu

Supplemental Material (Figures S1-S4)
Supplemental Table 1

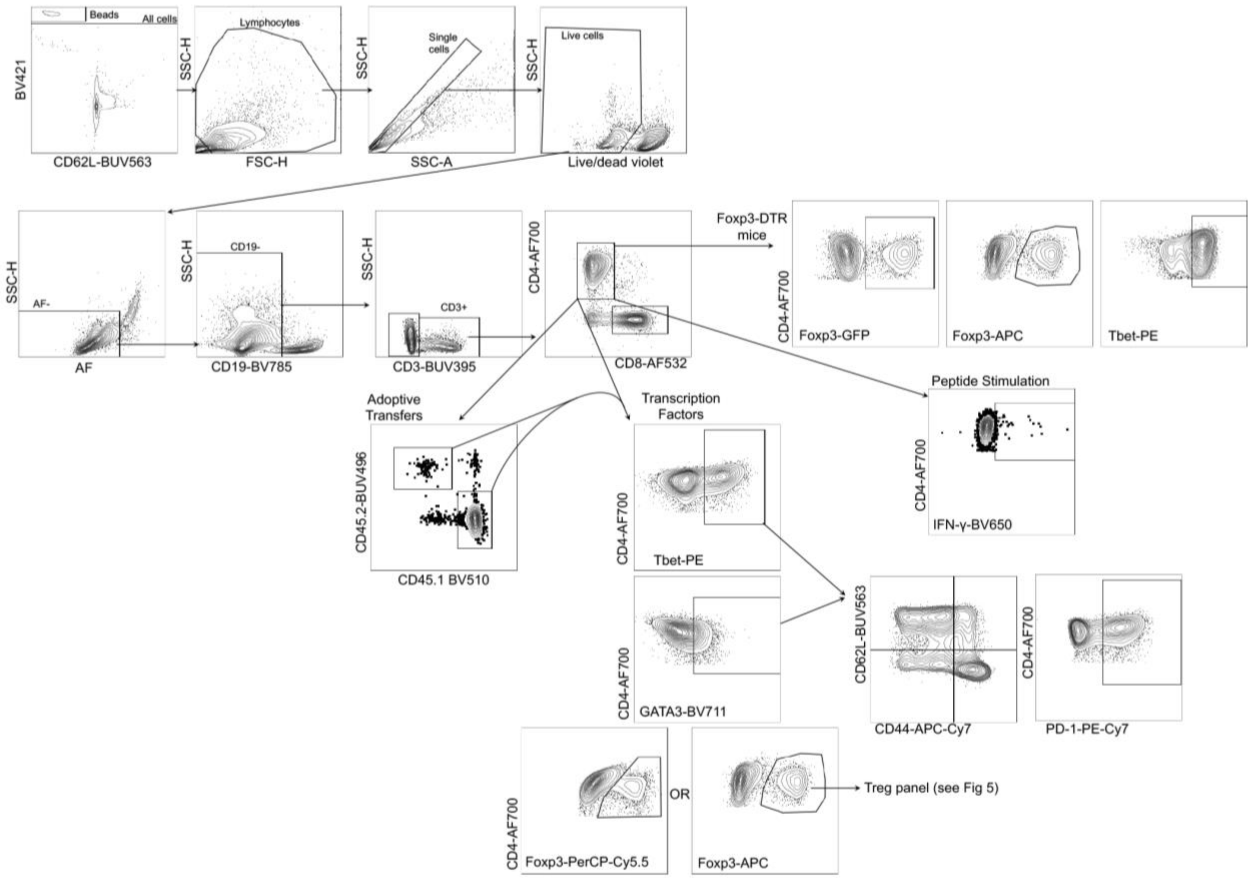

Supplemental Figure 1: Gating strategy for flow cytometry panels.

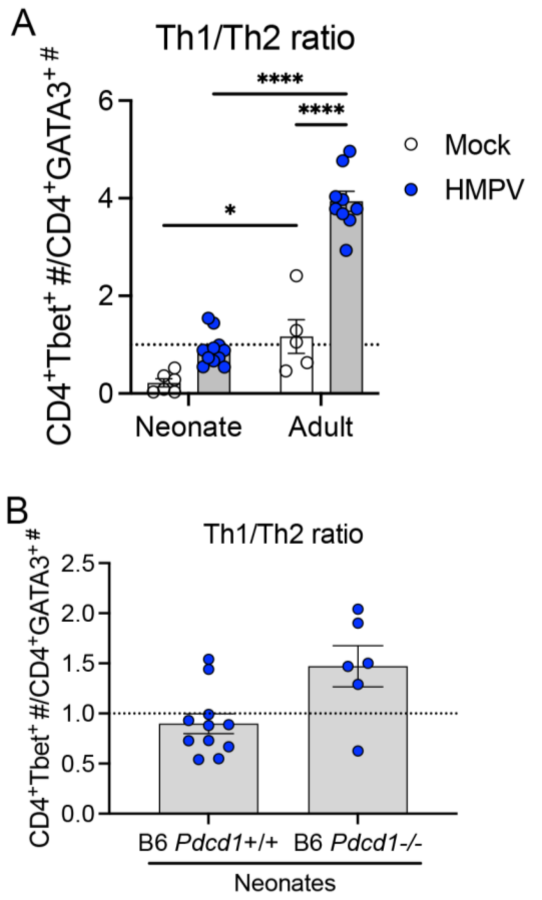

Supplemental Figure 2: Tbet/GATA3 ratio following HMPV infection. A.) Ratio of Tbet$^+$ CD4$^+$ T cells over GATA3$^+$ T cells in neonates or adult B6 mice following HMPV infection. Ratio of Th1/Th2 cells per individual animal. Neonate Mock n=6, Neonate HMPV n=11, Adult Mock n=5, Adult HMPV n=9; representative of 2-3 experiments. Statistical analysis performed via two-way ANOVA with Tukey's multiple comparisons, *p<0.05, ****p<0.0005. B.) Tbet/GATA3 ratio in HMPV-infected in B6 or *Pdcd1*$^{-/-}$ animals (n.s. by t-test).

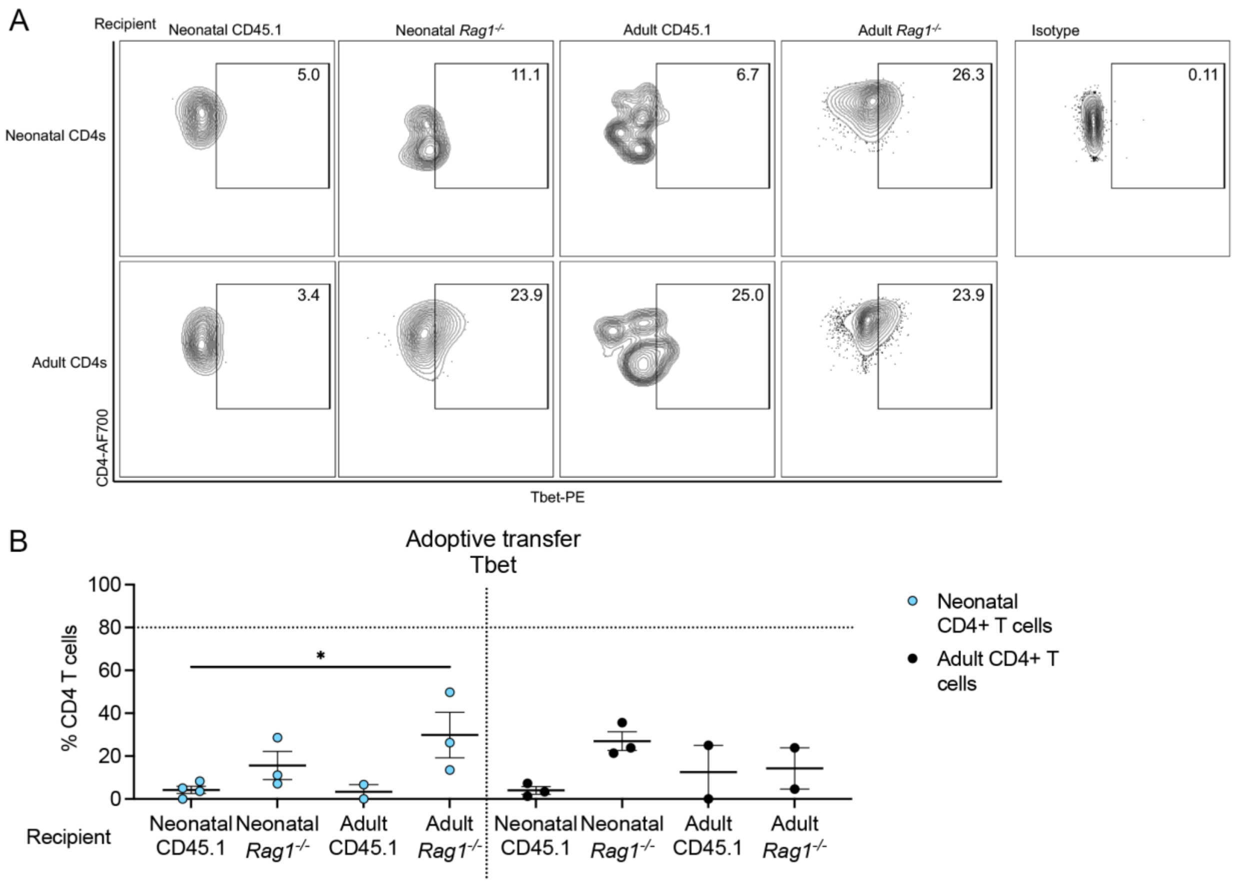

Supplemental Figure 3. Transfer of neonatal or adult donor CD4s in uninfected recipients showed limited homeostatic proliferation. Neonatal or adult CD4$^+$ T cells were transferred into CD45.1 congenically-marked or *Rag1*$^{-/-}$ neonates or adults in the absence of infection. After 7 days, T cells from the lung were assessed for Tbet positivity with limited induction of Th1s. *$p<0.05$ by two-way ANOVA with Tukey's multiple comparisons.

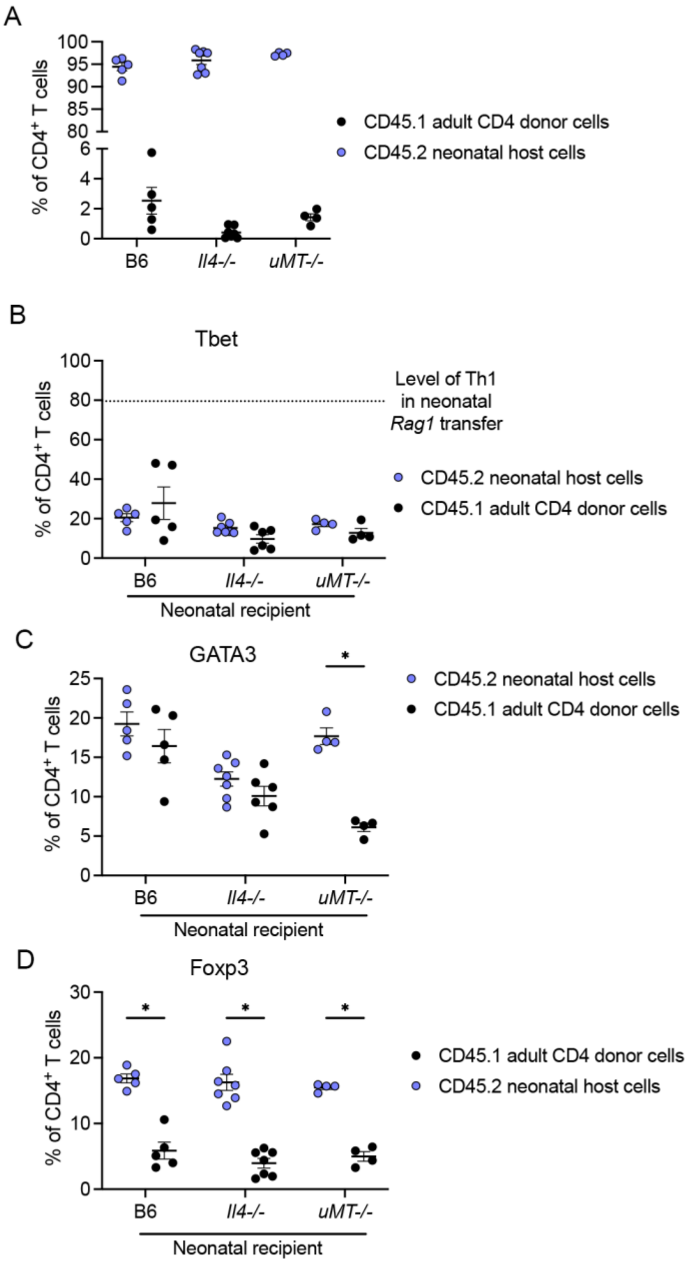

Supplemental Figure 4. **Suppression of Th1 induction does not require IL-4 or B cells**. A.) Chimerism of adult CD45.1 CD4^{+} donor T cells after neonatal transfer into CD45.2 hosts of varying genotypes (B6, *Il4*$^{-/-}$, and *μMT*$^{-/-}$) as assessed 7 days post-transfer and infection. n (from left to right): n=5, 5, 7, 6, 4, 4. B-D.) Tbet, GATA3, and Foxp3 expression in transferred adult cells compared to neonatal host cells in various genotypes. Th2 induction was reduced in *μMT*$^{-/-}$

mice while regulatory cells were more abundant in the neonatal host cells in all genotypes. * $p<0.05$ by multiple t-tests.

Supplemental Table 1. Reagents used for flow cytometry.

Surface markers:	Fluorophore	Species	Catalog Number	Clone
CD19	BV785	rat	115543	6D5
CD3e	BUV395	hamster	565992	145-2C11
CD4	AF700	rat	100536	RM4-5
CD44	APC-Cy7	rat	560568	IM7
CD62L	BUV563	rat	741230	MEL-14
CD8a	AF532	rat	58-0081-80	53-6.7
PD-1	PE-Cy7	rat	109110	RMP1-30
CD45.1	BV510	mouse	110741	A20
CD45.2	BUV496	mouse	741092	104
Transcription Factor staining				
T-bet	PE	mouse	644809	4B10
GATA-3	BV711	mouse	565449	L50-823
Foxp3	PerCP-Cy5.5	rat	45-5773-82	FJK-16s
Foxp3	APC	rat	17-5773-82	FJK-16s
Foxp3 (Foxp3-DTR mice only)	GFP			
Peptide stimulation plate				
IFNγ	BV650	rat	505831	XMG1.2
CD107a (LAMP1)	PE	rat	121611	1D4B
Treg panel				
CD25	AF488	rat	102017	PC61
CD39	AF647	rat	143808	Duha59
CD73	BV605	rat	127215	TY/11.8
TGF-β	PerCP-Cy5.5	mouse	141410	TW7-16B4
IL-10	BV510	rat	563277	JES5-16E3
